# Experiences among parents caring for children with juvenile idiopathic arthritis at a tertiary referral hospital in Kenya

**DOI:** 10.3389/fped.2025.1443529

**Published:** 2025-03-31

**Authors:** Hassan Silim, Susan Wamithi, Roselyter M. Riang’a, Angela Migowa

**Affiliations:** ^1^ Department of Paediatrics and Child Health, Aga Khan University Medical College East Africa, Nairobi, Kenya; ^2^ Department of Population Health, Aga Khan University Medical College East Africa, Nairobi, Kenya

**Keywords:** juvenile idiopathic arthritis, parental experiences, quality of life, Kenya, Africa

## Abstract

**Background:**

Juvenile idiopathic arthritis (JIA) is the most common rheumatic disease in childhood. Despite the availability of effective treatment strategies such as disease-modifying antirheumatic drugs (DMARDs), JIA is reported to have a negative impact on the quality of life of the patients and their caregivers. Ascertaining the perceptions of the parents will help facilitate more effective management strategies and improve outcomes for these patients. This study aimed to ascertain parental experiences and perceptions of caring for children with juvenile idiopathic arthritis (JIA).

**Methods:**

This was a single-center facility-based qualitative study. An in-depth interview guide was used to collect data from parents (*n* = 12) of children with juvenile idiopathic arthritis aged between 0 and 16 years attending the pediatric rheumatology clinic at a tertiary referral hospital in Kenya. Interviews were audio-recorded, transcribed verbatim, and thematically analyzed using the MAXQDA22.6 program.

**Results:**

A total of 12 parents participated in the study: 9 mothers and 3 fathers aged from 35 to 47 years. All parents were from middle- and low-income families. The study revealed parental experiences and perceptions in eight key domains: medical-related challenges, emotional aspects, coping mechanisms to deal with emotional burden, financial challenges, social challenges, healthcare personnel-associated experiences, disruption of work, and absenteeism by parents. Parents faced challenges in looking for resources and support to cope with the difficult moments of caring for their sick children. These challenges not only affected the parents but also affected the relationships in their entire families, including siblings, relatives, and their relationships among friends.

**Conclusion:**

The findings in this study highlight the challenges experienced by parents caring for children with JIA. Health workers need to be vigilant to the plight guardians go through and create support avenues to help them navigate the challenges they experience. There is a need for proper assessment of the physical and psychosocial well-being of parents and their families so that appropriate resources can be provided in promoting holistic patient-centered care.

## 1 Introduction

Juvenile idiopathic arthritis (JIA) is the most common rheumatic disease in childhood (1–3). The estimated global prevalence ranges from 3.8 to 400/100,000 with an incidence rate of 1.6–23/100,000 (4).

Despite the availability of effective treatment strategies such as disease-modifying antirheumatic drugs (DMARDs) and steroids, JIA is reported to have a negative impact on the quality of life of the patient and their caregivers/parents (3). There are often discrepancies between parents’ and healthcare workers' perspectives on disease control and disease status, and even in cases when JIA is considered well controlled by physicians, patients may feel otherwise (3, 5). Thus, ascertaining the perceptions of parents and guardians will help facilitate more effective management and improve outcomes for these patients (3).

The need for awareness and recognition of JIA is necessary, as illustrated in various studies in Africa (6, 7). These studies showed that the gap in expertise and lack of clinical capability to address the burden of JIA within low- to middle-income countries is challenging due to selection bias imposed by a dearth of pediatric rheumatology services and expertise (6). It has been postulated that environmental, genetic, cultural, and late presentation in the African population might contribute to the difference in epidemiology compared to high-income countries (8–10). It is reported that most of these patients are either missed or misdiagnosed. Misdiagnosis of these patients also means the physical, emotional, and financial burden that JIA poses on both the patient and their families (7). The differences noted in cultural and traditional beliefs and diagnostic capabilities in the healthcare facilities in Africa make the experiences of parents from high-income countries inapplicable to understanding the experiences of parents in the sub-Saharan setting.

### 1.1 Health-related quality of life among JIA patients

Most of the literature on the quality of life of children with JIA focused on quantitative assessment of health-related quality of life (HRQoL) (11). These quantitative studies generally reported poor or decreased quality of life in children suffering from JIA and their families (12, 13). A study in Egypt reported that 32 of 58 (55%) Egyptian children with JIA had suboptimal HRQoL (12). Similarly, 47%–57% of Dutch children with JIA experienced suboptimal HRQoL (13).

### 1.2 Family well-being among JIA patients

Families play an integral part in giving the child a sense of belonging. This makes the collaboration between the parents and health professionals an important pillar in the management of the sick child (14). Poor collaboration between the healthcare workers and the family leads to poor adherence to treatment, subjecting the family to emotional trauma and financial burden (14).

The lack of attention toward other children and the lack of communication between spouses created rifts among family members who felt either neglected or ignored (2, 15, 16).

### 1.3 Emotional and psychological experiences among JIA patients

Pain is one of the most challenging problems affecting JIA patients (16). Parents report that they felt helpless when their children were in pain and were affected psychologically and emotionally in trying to work out ways to try and alleviate the pain their children experienced (16).

A study done by Gómez-Ramírez et al. (17) reported complex mixed positive and negative emotions with remission and flare-ups that felt like a recurring roller-coaster ride. Stigma and loss of feeling of belonging in society also contributed to the poor mental health status of the families in this population (18, 19).

#### 1.3.1 Financial challenges

The cost of medications, such as DMARDs and biologics, caused a financial burden on families in sub-Saharan Africa (20, 21). The relatively high financial burden was associated with the disease's progress, the need for frequent admissions, and the need to travel long distances to attend clinics that were in other towns. Parents also had to take permission off work which resulted in pay cuts from their monthly income (3).

## 2 Study methods

### 2.1 Conceptual framework

**Figure 1:**
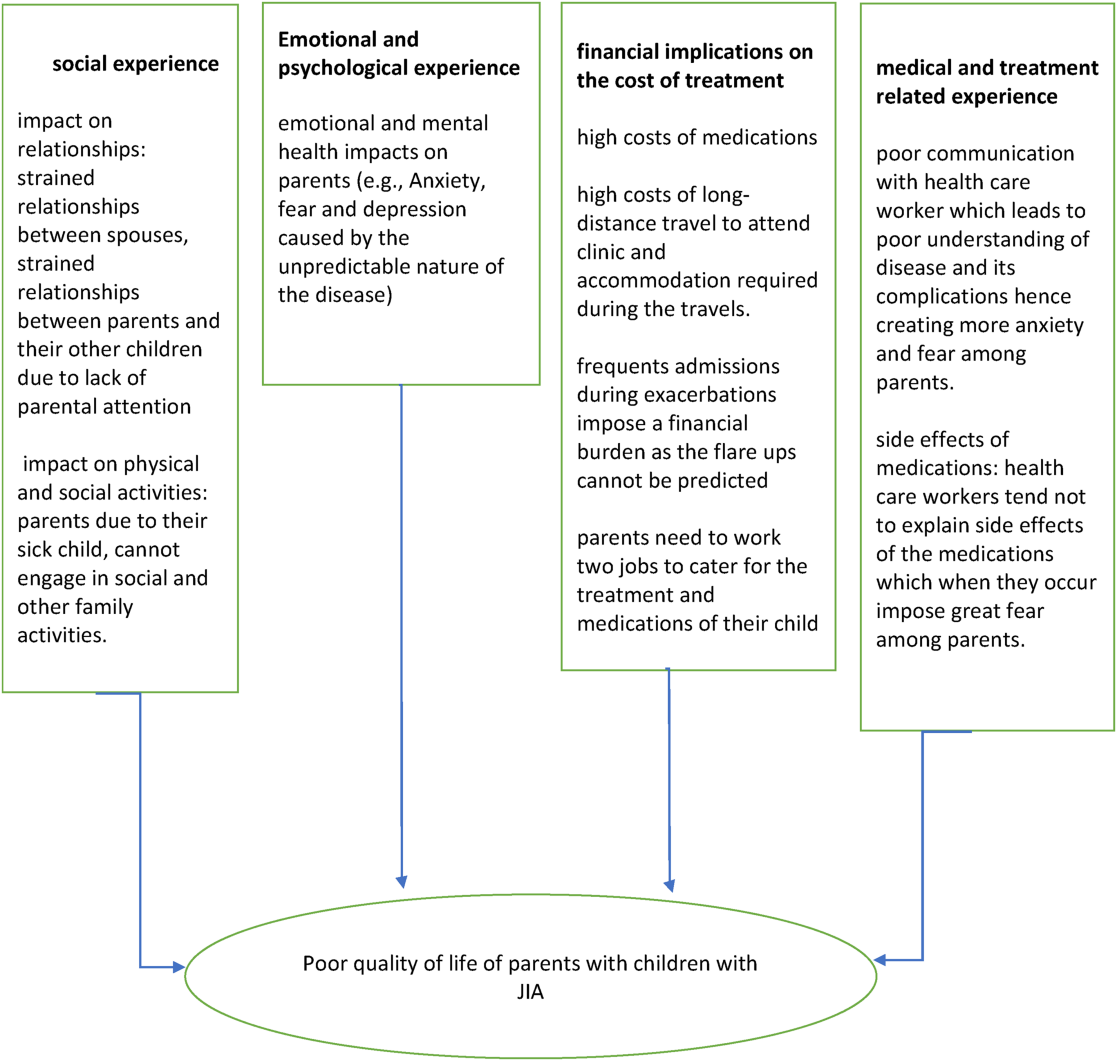
Conceptual framework among parents caring for children with JIA.

**Figure 2:**
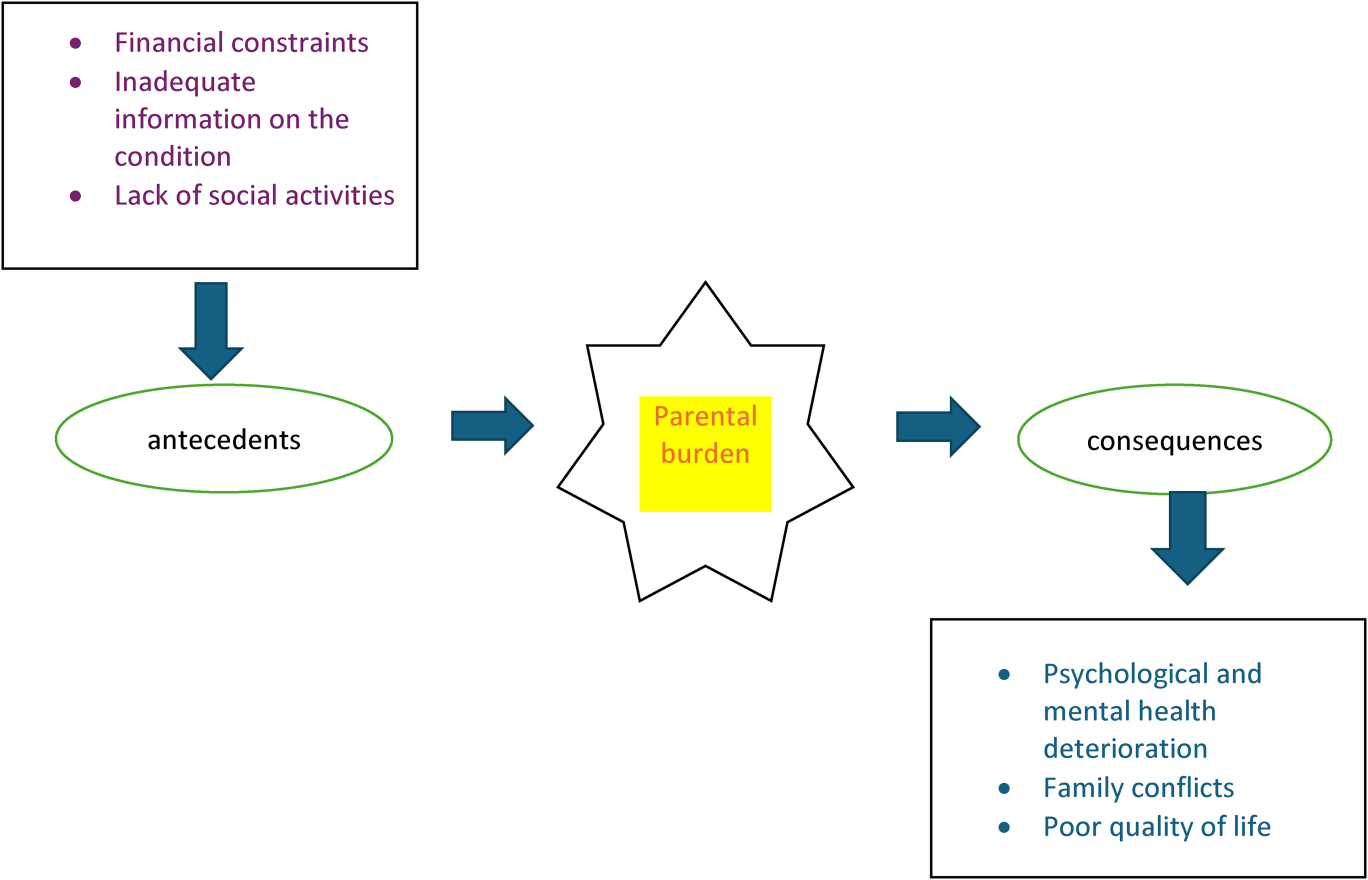
Contributing and consequences of parental burden.

Parental experiences were conceptualized using four major indicators: social experience, financial experiences, psychological experience, and medical and treatment-related experiences which impact their overall quality of life. These four concepts have been operationalized using various indicators as elaborated in Figures 1 and 2, which were retrieved from literature. These concepts guided the investigator to formulate the predetermined themes and sub-themes on which the data collection exercise was based on.

### 2.2 Study design

This was a phenomenological qualitative research design.

### 2.3 Study site

The study was conducted at the Aga Khan University Hospital (AKUH) in Nairobi, Kenya. AKUH is a non-governmental, urban-based, premier, tertiary, teaching, and referral healthcare facility. The hospital receives referrals for specialized medical care and diagnostic services from various hospitals and clinics within Kenya and other countries in the African region. The hospital is a 254-bed long-term care facility offering general medical services, specialist clinics, and diagnostic services in the field of rheumatology and others. AKUH was purposively selected among the six tertiary hospitals in Kenya because it is one of the only two hospitals with a qualified pediatric rheumatologist with a functioning rheumatology clinic that receives patients from East and Central Africa.

Data were collected from parents/caregivers of children with a diagnosis of juvenile idiopathic arthritis aged between 0 and 16 years attending the rheumatology clinic at AKUH. Any parent or guardian not conversant with Swahili or English was excluded.

The Department of Pediatric Rheumatology sees a total number of 18 patients from various countries in the sub-Saharan region. All patients who consented and were fluent in either the English or Swahili language were recruited, and the principal investigator worked closely with the head of the rheumatology department to identify potential participants from the Kenya Pediatric Rheumatology Registry (KAPRI) with a confirmed diagnosis of JIA. In total, 18 patients were identified in the registry at the time of the study. Twelve parents of the 18 identified were recruited, and 6 of the excluded participants were unreachable. All 12 patients who were from low- to middle-income families were interviewed to maximize reaching the data saturation threshold, despite the data saturation point being achieved by the 10th interview. We used criterion purposive sampling to provide rich information that gave an in-depth understanding of the caregivers' experiences and perspectives in caring for their children with JIA. A semi-structured interview guide was used to conduct the in-depth interviews. The interview guide was administered in English or Kiswahili languages depending on the respondent's language of preference. The translation was done by a certified translator at the Afro Lingo Translation Services in Nairobi. The development of interview guide questions was informed by the conceptual framework (Figure 1) which was developed from extensive literature review findings on the lived experiences of patients with various health conditions. The interview guide specifically collected information on the demographic characteristics of the respondents; social, economic, and health-related experiences of parents/caregivers; and how these experiences affected their quality of life/disease outcome. All the interviews were conducted in a quiet room at the health facility, which was convenient and social distance, and other COVID-19 preventive measures such as hand sanitizers and wearing of masks were adhered to. The first author (HS) recorded and transcribed the interviews and conducted the debrief that followed afterward.

### 2.4 Development of interview guide

The concepts in the framework also serve as predetermined themes guiding the deductive thematic data analysis. Open-ended questions were formulated in the interview guide by the first author following an extensive literature review on the quality of life of parents caring for children with JIA. It was later piloted to confirm the suitability of the questions during the interview sessions. Minimal changes in the framing of questions were made to avoid leading patients in their responses.

### 2.5 Data analysis

All the interviews were transcribed verbatim by the first author. Deductive thematic data analysis was conducted by the first author. Data analysis was informed by the predetermined themes presented in the conceptual framework (Figure 1) which was extracted from extensive literature review findings on the lived experiences of patients. The themes were developed by the first author and validated by qualitative research specialists. In the process of data analysis, we actively incorporated emerging themes while excluding those not supported by our data, culminating in the development of a revised conceptual framework, as illustrated in Table 1. The transcribed data were analyzed using the Max for Qualitative Data Analysis (MAXQDA) software program version 2022.6.

**Table 1:** Demographics of study participants.

No.	Sex	Age	Level of education
1	F	42	University graduate
2	F	46	University graduate
3	F	44	University graduate
4	M	35	University graduate
5	F	46	University graduate
6	F	42	University graduate
7	M	45	University graduate
8	F	43	University graduate
9	F	39	University graduate
10	F	47	Primary-level education
11	F	44	University graduate
12	M	42	Primary-level education

## 3 Results

### 3.1 Demographic characteristics of the study participants

Data were collected from twelve participants, of whom nine were female and three were male. Ten participants had attained a university level of education and two had acquired a primary level of education. The age of the participants ranged from 35 to 46 years (mean age, 42 years) as demonstrated in Table 1.

#### 3.1.1 Experiences among parents caring for children with juvenile idiopathic arthritis (JIA) at a tertiary referral hospital in Kenya

Various experiences of parents caring for children with juvenile idiopathic arthritis (JIA) at a tertiary referral hospital in Kenya were identified in the study, and these were grouped into eight main themes and several sub-themes as indicated in Table 2.

**Table 2:** Themes and sub-themes.

Themes	Sub-themes
1. Medical-related challenges	(a) Mystery of symptomatology.
	(b) Inefficient process at the health facility.
	(c) Treatment procedures.
	(d) Medication side effects.
2. Emotional and mental health challenges	(a) Struggles before diagnosis.
	(b) Struggles at the time of diagnosis.
	(c) Struggles after diagnosis.
3. Poor response to treatment, leading to developing coping mechanisms	
4. Financial challenges	(a) Cost of transport.
	(b) Cost of medications and diagnostics.
	(c) Struggles in meeting the special needs of their children.
	(d) Depletion of medical covers.
5. Disruption of work schedule and absenteeism	
6. Social challenges	(a) Relationships between relatives and friends.
	(b) Relationships between spouses.
	(c) Relationship with other children.
	(d) Relationships between siblings and cousins.
7. Healthcare personnel-associated issues	(a) Information from healthcare workers.
	(b) Lack of professionalism.
8. Preparing the child to accept and positively live with the condition	

#### 3.1.2 Perceptions of disease symptoms

This research sought to obtain perceptions and experiences faced by caregivers/parents in parenting children with JIA. From the study, it was identified that parents first noticed abnormal symptoms in their children at different ages ranging from 6 months up to when the child was 5 years old.

I think when the baby was one year two months that when the symptoms came, and the diagnosis came two years later. **Participant 3**

… The moment she turned 6 months old, when we started weaning, we noticed the swellings, so we took her to Nairobi Hospital, yeah so that's where we started our journey. **Participant 2**

The main symptoms that were first witnessed as reported by parents include swollen joints and headache. Other symptoms mentioned included fever and general body weakness.

…before we came out of conclusion that he has arthritis, he had some serious condition, he had a very spike fever, he had headache, he had body pain, joint pain, he had that condition. **IDI_JIA_05**

JIA subjected parents to various challenges ranging from medical-, emotional-, social-, financial-, and healthcare-related problems, absenteeism from work, healthcare personnel problems, and ways to cope with emotional burden as outlined below:

##### 3.1.2.1 Medical-related challenges

Various medical and health facility-related challenges emerged from the study ranging from inaccurate diagnosis, painful treatment procedures, long treatment process, and medication side effects.

###### 3.1.2.1.1 Perplexing symptoms

Caregivers demonstrated some challenges in knowing what their children were suffering from before formal diagnosis regarding the condition was conveyed to them. The participants narrated the journey before diagnosis as being difficult, tough, and confused with other diseases.

At first she was tested for Lyme disease because it runs where we were from and uhm… in England Lyme disease is everywhere and her aunt she was diagnosed with Lyme disease for a very long time, and I did not want that to happen to my daughter. **Participant 2**

Okay what we thought was maybe she could be having any allergy because that was the time we started weaning, so we thought that whatever we are feeding her could trigger the swellings. **Participant 7**

Healthcare personnel took a long time to diagnose JIA and occasionally misdiagnosed patients with other medical conditions.

So, at first, I went to the hospital, the doctor who saw us thought it was tonsillitis. We were given some medication that we started, but by the third day, the fevers were still persistent, I decided to go back. **Participant 10**

###### 3.1.2.1.2 Long processes at the health facilities

Parents had their schedules disrupted due to the long waiting time at the clinics and at the laboratory during the healthcare visits at the facility. They felt that no one understood their plight and what they were going through.

Every trip to the hospital ends up 5 hours late, even if you think it will be shorter (laughs, we had to go so many times to hospital for preliminary things that I thought were unnecessary. Nothing ever runs smoothly there. Yeah, so I think that has been really… really challenging. **Participant 6**

###### 3.1.2.1.3 Painful treatment procedures

Parents considered administering injections to their kids as nerve-wracking. They could not bear the thought of inflicting pain on their already ailing child. Some parents opted to find alternative treatment regimens to try and not inflict pain on their children.

The first time I felt like it was very unfair. You know, why her? Was it worth it? Is there an easier way out? … I can inject because my pain tolerance is a bit high, but I chose not to do it to her and uhm… the dad took it up because he was the best person to do it. **Participant 1**

…… there were times to be consistent with how I inject her. Sometimes she bleeds, and I feel guilty. **Participant 2**

###### 3.1.2.1.4 Medication side effects

Medication side effects were a major problem for parents. Some of the side effects were concerning to parents, e.g., weight gain, frequent urination, and change of skin color; because of the side effects, parents opted to stop treatment and use traditional unproven treatment methods.

They are of good help, although sometimes there are worrying maybe the side effects, but they are fine, she normally undergoes various tests, our worry is just the side effects…Our worry is if she takes too much of drugs, there could be side effects. **Participant 7**

I get really frustrated. After taking her medication, she cannot stay close to 15 min. She must ask for permission to visit the restroom. It makes her hate herself especially when asked by other students why she keeps visiting the restroom all the time. **Participant 9**

In desperation to help their children, some parents turned to using other traditional methods of treatment. Some were ready to break the law by hunting state-protected, endangered animals, e.g., pangolins, to obtain their scales which they believed could help cure the condition. One parent decided to take her child to undergo “hijama” (cupping) which she had been advised would be helpful, despite the risk of her child getting infections as the procedure is usually performed with unsterilized equipment.

…There is this animal called pangolin…According to people they say this animal is a very expensive animal in Kenya and worldwide. If you are found with this animal, you are likely to be arrested and charged…I don’t know if the scale of this animal treats arthritis. But the way I have seen it if you google it treats …pangolin scales treat arthritis. Participant 10

There was a time I happened to take her to do ‘Hijama’, that was in Kenya, when she said she did not feel like that drug is working. It was when the arthritis started to be a bit problematic, so I decided to do Hijama and stop any other medication. IDI_JIA_10

##### 3.1.2.2 Emotional challenges

In the study, it was identified that JIA emotionally affected caregivers. Emotional challenges existed at various stages of the disease ranging from before diagnosis, during treatment of the condition, and after diagnosis as discussed below.

###### 3.1.2.2.1 Struggles before diagnosis

It was noted that it generally took a long time before the right diagnosis was made. As a result, the uncertainty about the condition caused emotional and physical distress to caregivers, leading to stress and depression. Moreover, one caregiver was worried about the condition and noted that it was only Allah who could heal the child. Some felt embarrassed when visiting the hospital as even the casual workers would recognize them due to the frequent visits and admissions.

Trust me it wasn't an easy moment, I lost weight, I was emotionally disturbed, I was physically disturbed, you know even people will forget your name they are like the mother of that sick, sick child because we were always at the hospital, until even I kept telling people I was in hospital, it was such a devastating experience, because even in hospital like I remember while in xxx hospital even the cleaners knew my name mama XXXX… you know, you've gone there and they are like ooh you are back? it wasn't an easy journey it was very tough for me. **Participant 3**

Before we came to the diagnosis, we went through a lot of difficulties…. am a Muslim we believe that each condition comes from the Almighty God. So, and maybe that condition only Allah will normally cure but, we normally treat but that condition can only be cured by the help and prayer for Allah, that's my thought. **Participant 5**

###### 3.1.2.2.2 Struggles at the time of diagnosis

Several caregivers mentioned that they were shocked to learn that the child had JIA. They described it as the hardest thing they had ever experienced. They were worried about the condition and had many questions as to why such a rare condition affected their child. They were particularly worried about the chronic nature of the condition and what the future holds in terms of the progression of the disease.

Exhausting and challenging I don't have the right words, but it had been the hardest thing we have ever had to do in our lives, and we are still doing it. **Participant 1**

Poor knowledge about JIA among parents and the thoughts of associating arthritis with older people contributed to parental distress. They also related JIA with permanent disability.

Normally, my perception was arthritis normally attacks old age, it is a condition maybe cripples you to be bedridden, maybe, that condition cannot be treated earlier. So, when he was diagnosed with that, I was a bit worried about the condition and I asked a lot of questions, is it a condition that we can get out of it. **Participant 5**

###### 3.1.2.2.3 Struggles after diagnosis

After diagnosis, the caregivers were scared and anxious while managing JIA. It was also difficult for the parents to accept that JIA is a chronic condition. Some of the caretakers expressed sadness, desperation, helplessness, and stress from seeing the child suffer from JIA and not being able to do anything about it.

Desperation, because you are helpless. You don't know what to do. The child is crying at night and the painkillers are not helping. It's a feeling of hopelessness because there is nothing much you can do about it except praying that she feels better. **Participant 1**

In addition, the child with JIA is seen as a burden to others. One parent noted that he would frequently miss work and could n't concentrate on the tasks due to anxiety and stress.

At times I would take permission at times I would skip work. So, since that time up to now… I was disturbed emotionally. When you see a child of her age. **Participant 8**

##### 3.1.2.3 Poor response to treatment, leading to developing coping mechanisms

Due to the emotional roller coaster, and not seeing the end of the journey despite treatment, parents decided to turn to their spiritual beliefs and to leave everything to God, as they believed that God is the healer and can cure anything.

You see, that is something that discourages, I mean discouraging, after somebody taking drugs for two years plus but there is nothing, there is no improvement… You know doctor, when it reaches that scenario, you know you just leave everything to God… you just leave everything to God and say it’s God who created and it is God also who takes. **Participant 11**

Moreover, there was a need for an opportunity for parents and caregivers to share experiences. This was through the establishment of support groups for caregivers. It was further recommended that healthcare providers need to offer and improve their support system to the caregivers or parents.

Also, for the caregivers, there is no support system at all. At the end of the day, it is an outpatient kind of condition. We are the ones who see this child, take care of them but there is no support for us. I feel the doctors should uhm… I don't know… make sure we have a support system. **Participant 1**

Some parents recommended support groups to deal with emotional burden, as they felt knowing what other parents are going through and being able to relate to what they are going through will be helpful to their well-being.

I needed other parents that have had this already and what they did. I needed to talk to them. Even if new parents have access to that system even temporarily. It's key…. If you have other parents that you can share those awful stories with it helps. **Participant 2**

##### 3.1.2.4 Financial challenges

###### 3.1.2.4.1 Cost of transport

High cost of transport and long distance to the facility were found as a main challenge in accessing the hospital for clinic visits regularly. Some caregivers mentioned that due to the unpredictable course of the condition, there was even a feeling of need to relocate for easy access to the health facility.

I had challenges because even traveling from ******to *****was a problem. I stayed far from the bus stop. I lived in the suburbs, so I only had the option of using a taxi as there were no motorbikes then…. **Participant 9**

###### 3.1.2.4.2 Cost of treatment

The expensive cost of treatment and medication was identified as one of the main challenges. Most parents could not appreciate that the treatment and medications could be so expensive due to the complexity and chronic nature of the condition. Parents had to feel the pinch of expensive diagnostic tests that would cost huge sums of money before even buying medications which were also noted to be quite expensive. The expensive nature of the medicines and the laboratory test depleted their medical covers in a short time and parents later had to go back to their pockets to sustain the treatment regimen.

I don't have a medical card, so the little I have I am going to spend direct for transportation for medical, now that. **Participant 11**

…. It's a quite expensive treatment that I have never come across, it is costly and at some point, I am insured I have a medical insurance, at some point it gets out of control maybe your limit is passed. **Participant 5**

###### 3.1.2.4.3 Struggles in meeting the special needs of their child

Lack of money by some parents to provide for the child's special needs, such as good schools and seeking financial support from relatives and friends, was also seen as a burden to other people. One parent portrayed distress due to the inability to take her to a private school that was close to their home. Instead, the child had to travel long distances to school, which would affect the child's well-being.

My baby's condition really stresses me out. I have no idea of what to do…I get very frustrated. If I had money, she would not have attended her current school…It is very far. I could have enrolled her in a private school with a school bus, but I don't have the ability to do so. **Participant 9**

###### 3.1.2.4.4 Depletion of medical covers

In addition, having medical cover was noted as a privilege since the cost of treatment and medication was high and sometimes unaffordable when paid out of cash. One caregiver developed a rapport with people at the National Health Insurance Fund (NHIF) to help add extra money to the medical coverage limit.

Also because of the financial part of it I …we also have a medical cover, by the way, were it not for my employer I don't know how I would have managed this condition, (laughs) in terms of costs. **Participant 4**

For financial challenges, I just developed rapport with people at NHIF because I think they have that window of adding some extra money, so that's what I did…. **Participant 3**

##### 3.1.2.5 Disruption of work schedule and absenteeism

The study noted that the diagnosis had impacted one's daily routine income-generating activities. A lot of time was taken by the need to attend to the child with JIA. Most of the time was spent at the hospital during the clinic appointments and needed for further investigations into the condition.

It was just by God's grace that my boss understood me, you see, if my boss did not understand me, trust me today I wouldn't be working, because you are admitted for one week, the following week is medication, in between the baby gets sick, you get admitted again …. **Participant 3**

Some of the caregivers were able to have flexible work schedules depending on the type of work. Others received good support and understanding from the work supervisors despite the number of missed days from work trying to attend to the sick child.

I mean I have been able to have flexible schedule because I have explained to them. With my husband, his work is a bit less flexible and a lot more challenging. **Participant 6**

It was noted that absenteeism and the need to accommodate the demands of caring for the child with JIA resulted in poor work output. One parent who had a supervisory role in a company had to leave her job due to the challenges she faced in trying to accommodate clinic visits which in turn led to failure to perform her duties as a manager.

It was tough. Especially at that time when she was first diagnosed… my work was not flexible…Yes, so it was tough having to take some time off because I ran the whole floor. Suddenly, I had to take some time off. It was uhm… I can't lie… it was really rough…which then led to me actually leaving that job. **Participant 2**

##### 3.1.2.6 Social challenges

###### 3.1.2.6.1 Impact on relationships with relatives and friends

There was a belief among close relatives and friends that JIA was hereditary. Some relatives superstitiously believed that the child could have been bewitched resulting in stigmatization, e.g., being excluded from family gatherings and being seen as a bother to relatives, discouraging feedback and comments from relatives.

To think that somebody bewitched your child…you know…At times it would make sense but uhm… because we are Christians, we would pray about it and uhm… we chose to research about it. It wasn't still not making sense to us, the knowledge we acquired from reading helped us see, pass all that and take the medication seriously. **Participant 1**

Maybe the negative part of it now is the relatives, they could not understand why XXXX was the way she was, but we had to sit them down and talk to them and tell them they also needed to support XXXX, because it is not her wish that she is the way she is. **Participant 4**

Yes, we did because the minute you mention your child cannot do one, two, and three, obviously, you are not included in most of the activities because they think that you have a lot of issues around you… you know… They know you are not doing well financially, when they see you, they just think you are just about to ask for some money. **Participant 1**

There was also a notable positive impact on the relationship between the caregivers and their relatives. Some of the caregivers received encouragement and support after the diagnosis of the child with JIA. They also received financial support while others showed sympathy for the child and the family.

They helped me when I went to get an NHIF card. That is because I would use a lot of money, so, the card would come in handy to help in that…They gave me hope and told me that because the baby was very young, she would get better and recover. They would call me after I go for the clinic and ask how everything went. **Participant 9**

The caregivers reported that the bonds between relatives and family members grew stronger. Some of the relatives would be there to help the family in instances where the family of the sick child needed help with the cost of treatment.

We all find a way on how we can make her feel better, so for example right now we…we have a brother who is working with Save the children, his family offered to include my daughter to their medical card, generally to support our family. **Participant 11**

###### 3.1.2.6.2 Spousal relationship after JIA diagnosis

The relationships between spouses were generally strengthened by their child's conditions. However, some parents also reported that their relationship was damaged in the process of caring for their sick child.

I think because of financial constraint we realized we don't have any other person but us. I think we held on to each other because we did not have anyone else out there. We drew stronger because we had no one else. As much as it was strained, it was just the emotional bit. **Participant 1**

On the other hand, the condition also presented challenges to the spouses. One caregiver noted that there were different levels of understanding medical terms and conditions which led to rifts and arguments between them.

Yes, it did but uhm…it's not so much about the relatives and friends, it was to do with our personality and coping mechanisms. We are different. Wired differently and our coping mechanisms were quite different. So that was a bit challenging. **Participant 1**

###### 3.1.2.6.3 Relationship with other children

“Attending to the child with JIA led to reduced time spent with other siblings.” This led to the feeling that the baby with JIA was loved and given more attention. For instance, one caregiver noted that one of the children felt left out from being given medicines.

…My son felt like I love my daughter more than I love him (Laughs) you know, he could not understand why he was being left at home when I took my daughter to the hospital. At some point he asked, ‘Mum, why is it that XXXX comes with you?’ He feels like you love the other baby more than him, which is not the case, definitely you love all your children. **Participant 3**

###### 3.1.2.6.4 Relationship between siblings and cousins

The study noted that the relationship between siblings after the child was diagnosed with JIA varied. Although most were noted to be supportive and developed empathy and pity toward the child with JIA, they also were confused and found it difficult to understand why their sibling suffered from this condition. They were also devastated by the limited interaction with their siblings for fear of the sick sibling getting infections due to depressed immunity. Most of them would help their sick sibling with chores such as washing her undergarments, tying shoelaces, and putting on her clothes.

Yeah, you know like the girls share a bedroom, they would notice maybe when she is in pain and they would come in handy, they would help her maybe put on her clothes, tie the shoelaces, maybe help her wash her undergarments. Those kinds of chores that a girl would do to support and even now they still do. **Participant 4**

##### 3.1.2.7 Healthcare personnel-associated issues

It was noted that the available healthcare providers were professional and offered specialized services. They were described as respectful and supportive to the parents of a child with JIA, hence making the caregivers able to learn a lot about the condition.

I can confidently say that the medication she is taking has helped because, as I had told you it's not like before, every other month she had to be admitted, every other week we had to be at the hospital because of fevers. This has seized. **Participant 3**

###### 3.1.2.7.1 Information from healthcare workers

The caregivers noted that they received adequate information about JIA from the healthcare providers. They received encouragement and social support from healthcare providers. In addition, their questions on JIA were well addressed by the healthcare providers. Similarly, one caregiver noted that in the USA she was able to receive adequate information about JIA and got connected to the peer support system.

We had a very good doctor; Dr. xxx I think is one of the best doctors in the country. **Participant 4**

Dr. xxx she was so supportive, she really educated us, she even gave us some pamphlets to go and read o that we can be more conversant, she explained the side effects, what to expect, the dos and the don'ts with the steroids, so we were contented. **Participant 10**

In the US they really give you all the information, the pamphlets…the support system…. **Participant 2**

On the other hand, some of the caregivers noted that they received inadequate information. They therefore needed more information about the JIA condition and hence had to do more research by themselves.

I don't think the information is sufficient the one we were given…, In my case, I felt that I needed to research more because at the end of the day. **Participant 1**

###### 3.1.2.7.2 Lack of professionalism

Other experiences included the view and observation that some of the healthcare providers were like in business at the expense of patients, especially before the real diagnosis of the condition. One parent noted that some doctors would do unnecessary procedures which they felt was just a way of taking advantage of the desperation to find a solution for their sick child.

Like for example, the surgeries, the doctor that did the surgery was not a young doctor, you know, outside here the world will tell you go to the old people, they are well versed in medication or in the medical field. But he chose to do surgery for the baby, which was very unnecessary So, for me I feel most of the doctors today are just trying to make money from patients, and from naivety of patients… they are not ready to refer patients to another doctor even if they know that they cannot treat that condition, they still want to treat that condition. **Participant 1**

Some parents noted that some doctors lacked communication skills and felt that they were at times rude. It was noted that despite the long journey parents would endure attending clinics, the doctors never took time to explain the situation of their child during that visit, which made them feel unwelcome and unsatisfied with the services.

All these experiences I am telling you… there are some doctors I met, but they are not many. Where you go to see the doctor and you are not even told to welcome and have a seat. They just stare at you. There is a doctor I met, I will not mention the name and the hospital but (pause) you feel bad because you are looking for assistance and you have traveled all the way to seek the doctors' opinion… what they can minimally do for you, is just to explain. **Participant 4**

##### 3.1.2.8 Preparing the child to accept and positively live with the condition

It was noted that parents were devastated by the fact that their children could n't engage in physical activities like any other normal child. It was also noted that due to the pain experienced by the children, some parents developed ways to psychologically strengthen the children through the pain. Parents would try engaging their sick child in their favorite activity, e.g., some would take their child shopping and horse riding just to try and deflect the thought of their child being in pain.

There are times XXXX would be affected emotionally, her self-esteem goes down because she cannot function like any other normal child outside there even playing. She would not run, she would not be doing those activities the other kids may be doing, so somehow it really affected us and affected her, but we encouraged her to and gave her hope to try and do whatever she wants we it is possible. **Participant 4**

## 4 Discussion

The study revealed that parents encountered several challenges from the time of diagnosis and throughout the journey of the disease. Initially, parents struggled with the bizarre, unusual course of symptoms experienced by the children. They often believed that their children were suffering from conditions such as Lyme disease. Some felt that their condition was a result of strenuous activity. They struggled from not being familiar with the disease, not being able to help their children during pain, struggles with healthcare systems, lack of information from the healthcare givers, and medication side effects. These struggles impacted the emotional well-being of parents and their families.

Findings in this study expand our understanding of the eight areas parents particularly experienced struggles namely:

(1) Social challenges (marital relationships, relationship between siblings, relationship with relatives and friends)
(2) Emotional challenges
(3) Financial challenges
(4) Healthcare personnel-related challenges
(5) Medical-related challenges
(6) Absenteeism from work
(7) Coping mechanisms
(8) Positive psychological reinforcement

### 4.1 Social challenges

A study by Yuwen et al. “Struggling in the dark to help my child” noted that parents experienced challenges with their spouses and other children and that the condition negatively impacted their relationships (16). In our study, however, it is noted that the relationships between spouses were mostly positively impacted by the condition. Parents became closer in terms of communication and developed a common ground in trying to help their children through the tough journey of dealing with JIA. This study also showed that relationships were mostly strengthened between siblings with increased empathy for their sick sibling in contrast to what was reported in a study “Having a sibling with JIA” by Jones et al. which showed that lack of attention toward other children created rifts between family members (15, 22).

### 4.2 Emotional challenges

Parental stress was attributed to poor outcomes in managing patients with JIA in a study by Caes et al. (23). Parental catastrophizing about child pain was shown to be a restricting factor in engagement in physical activities in children with JIA (23). In our study, parents played a pivotal role in developing coping mechanisms to deal with the pain in their children. Parents use religious and spiritual ways to encourage their children to get through the tough times in the course of the disease. Parents try to encourage children to take part in activities they enjoy, for example, one mother will go horse riding with her child to help her feel normal and enjoy herself.

Similar to our study findings, Gómez-Ramírez et al. in a study titled “A recurring roller coaster,” found that parents experienced mixed emotions and despair in their child's inability to participate in physical activities and inability to accept the diagnosis initially as most felt that the condition mostly affected the elderly (17). In the same study by Gómez-Ramírez et al., it was shown that improving emotional support for parents improves psychosocial outcomes and well-being of children with JIA (17). Hence, awareness of this complex emotional status of parents caring for children with JIA will help healthcare workers offer better support for parents and result in better patient outcomes.

Similar findings as studies above in the literature review noted that procedures that parents had to do to administer injectable medications to their children were nerve-breaking and hard.

They felt that they were contributing more pain to their children already suffering the burden of JIA (22, 24).

As reported in the study “The ‘medical career’ experienced by mothers of a child diagnosed with juvenile idiopathic arthritis” by Waite et al, our study showed similar findings to most of the studies quoted in the literature review that parents could not bear seeing their children suffering from pain and that they felt helpless and devastated (15, 24).

Our study revealed that parents, in desperation to help alleviate suffering from their children, sometimes opted for traditional non-proven interventions. One father reported that he was informed that JIA could be treated using the scales of the pangolin which is illegal to hunt and kill within the region. This was due to the strong cultural and religious beliefs of societies within our region.

### 4.3 Financial burden

Studies done in high-income countries showed a huge financial burden on families in terms of the cost of medications and the cost of other modalities of treatment such as physiotherapy (25). Studies in India and Canada showed lower costs of treatment in the management of JIA, while the costs in the USA and Europe were consistently higher (25). One article from a low- to middle-income country showed considerably lower costs compared to other regions. It was attributed to lower living expenses and lower access to biological treatment (25). It was noted in our study that the cost of transport to healthcare facilities bore a huge financial burden on parents. Some lived in the suburbs where there were no buses and motorbikes, which necessitated them hiring taxis to reach the healthcare facilities. Parents also reported expensive tests and high doctors' fees charged during admissions. Hence, more studies are needed within our region to reflect the actual financial burden JIA has on our societies.

### 4.4 Challenges with healthcare personnel

Communication between parents and healthcare workers is key in helping parents and the child to understand the disease course and outcomes which greatly helps reduce parental anxiety and distress (1). Barlow et al. studied the perception of psycho-educational intervention in JIA and found that parents could not get sufficient information from healthcare workers regarding JIA and often opted to read articles online which sometimes were outdated. This study showed mixed reactions from parents when it came to getting information regarding the condition from healthcare workers (1). In our study, most parents were satisfied with the amount of information provided by the healthcare workers (1). They noted that healthcare workers dedicated enough time to trying to explain the condition, plan of treatment, and side effects of medications. Nonetheless, parents in our study noted that some healthcare practitioners lacked empathy and professionalism and felt that communication was not adequate.

### 4.5 Medical-related challenges

One other theme noted in this study was that several parents reported frustrations with the hospital systems, i.e., the queuing system and patient services. Most patients felt that they wasted most of their time and energy in trying to pass through the processes needed to reach the doctor. They reported frustration and devastation due to the long waiting times encountered in the hospitals. However, they reported excellent services when they entered the doctor's room.

### 4.6 Absenteeism from work

In our study, it was noted that parents missed work and even had to quit their jobs to care for their sick children. One mother in our study noted that the demanding nature of her job resulted in her not being able to perform her duties and meet deadlines because of the need to constantly care for the child during her flare-ups and attendance at the child's clinics. One father in our study noted that he had to take time off regularly to attend clinics as the mother had other young children who she needed also to care for. In contrast to a study by McNeill (26), fathers hardly missed work, as it was noted that they took work as an escape route from the challenges they experienced in the management of the child with JIA. It was noted by McNeill that one father who was unemployed engaged in household work most of the day to try and forget the challenges they experienced (26).

### 4.7 Coping mechanisms and positive psychological reinforcement

Parents play a big role in promoting pain coping mechanisms among their sick children. In our study, parents tried to encourage their children and not give them false hope that the condition would be cured as they grow older. They did this to help condition their minds and bodies to tolerate the pain as they knew JIA was a chronic condition and they would need to cope with its effects for most of their lives. Our findings are in contrast to a study done by Palermo (27) which showed most children with chronic illnesses tended to miss school and avoid physical activities because their parents discouraged them from engaging in or attending school activities. This contributed to more anxiety for the children when they experienced pain as they felt that something bad or horrible was about to happen as they had been conditioned to believe so (27). Caes et al. and Feinstein et al. found similar findings to the study done by Palermo which suggested that parental psychological inflexibility and catastrophizing of pain led to higher levels of anxiety among children and their parents and also promoted disability among children with JIA (23, 28).

This study's qualitative nature described the parents' experiences, thus rich in data that will be useful in forming healthcare policies to guide healthcare workers in improving the experiences of parents caring for children with JIA. Participants responded to all the interviewers' questions, thus enriching the information gathered and making it possible to draw relevant conclusions.

#### 4.7.1 Strengths

The study was conducted in the preferred language of the patient (English and Swahili). This helped gain a better depth of the challenges faced by parents. The qualitative nature of the study gives the actual sense and derives an in-depth understanding of the exact challenges parents go through in terms of finances, socioemotional well-being, etc.

#### 4.7.2 Limitations

Patients from only one center were interviewed for the study; therefore, the study may lack insights from patients' parents in other centers. We did not get to interview the child, which would have given the study a better insight into the challenges faced by the families. The small number of participants might have underestimated the real burden of challenges faced by parents; hence, further studies are encouraged to look at the topic.

## 5 Conclusion

The parents of children with JIA included in this study reported complex mixed positive and negative emotions that had an impact on their well-being. Healthcare providers caring for children with JIA should be aware of these complex emotional experiences to better support parents and help improve JIA outcomes. Religious and cultural beliefs tend to play a vital role in how parents cope with the effects of JIA. Relationships between parents and their spouses as well as their relationships with other children were often positively impacted by JIA.

### 5.1 Recommendations

#### 5.1.1 Recommendations to researchers

Further research in low- to middle-income countries is required to determine further challenges experienced by parents.

There is a need to train and sensitize health professionals on rheumatological conditions to mitigate the negative effects conditions such as JIA have on families and improve communication between parents and healthcare workers to come up with improved support systems for families.

#### 5.1.2 Recommendations to policymakers

Government policies should focus on establishing avenues to subsidize the cost of medications to ease the financial burden on parents. Establishing government-funded programs would provide specialized services cushion to parents to help them alleviate the need to pay high fees in private hospitals. There is a need to establish government-led campaigns to be sensitive to the society of rheumatologic and other non-communicable conditions to deal with stigma.

## Data Availability

The original contributions presented in the study are included in the article/Supplementary Material; further inquiries can be directed to the corresponding author.
